# Outcomes and future activities of the ‘Pan-European network in Lipidomics and EpiLipidomics – EpiLipidNET’

**DOI:** 10.1007/s11306-026-02396-7

**Published:** 2026-03-11

**Authors:** Julia Kuligowski, Justine Bertrand-Michel, Amaury Cazenave-Gassiot, Laura Goracci, Pedro Domingues, Matej Orešič, Margret Thorsteinsdottir, Florian Gruber, Egon L. Willighagen, Corinne M. Spickett, Tatjana Ruskovska, Valerie B. O’Donnell, Patricia Prabutzki, Snježana Petrović, Joanna Godzien, Sara Tortorella, Ana Reis, Irundika H. K. Dias, Guy Schleyer, Rosário Domingues, Maria Fedorova

**Affiliations:** 1 Spanish Network in Maternal, Neonatal, Child and Developmental Health Research (RICORS- SAMID) (RD24/0013/0014), Neonatal Research Group, Health Research Institute La Fe, Avenida Fernando Abril Martorell 106, Valencia, 46026 Spain; 2 Servicio de Análisis de Vesículas Extracelulares (SAVE), Health Research Institute La Fe, Avda Fernando Abril Martorell 106, 46026 Valencia, Spain; 3 MetaboHUB-MetaToul, National Infrastructure of Metabolomics and Fluxomics, 31077 Toulouse, France; 4 Université de Toulouse, Inserm, I2MC, 31 432 Toulouse, France; 5 Department of Biochemistry and Precision Medicine TRP, Yong Loo Lin School of Medicine, National University of Singapore, Singapore, 117456 Singapore; 6 DAISY Lab, Department of Chemistry, Biology and Biotechnology, University of Perugia, Via dell’ Elce di Sotto 8, Perugia, 06123 Italy; 7 Mass Spectrometry Center, Department of Chemistry, LAQV-REQUIMTE, University of Aveiro, Campus Universitário de Santiago, Aveiro, 3810-193 Portugal; 8 School of Medical Sciences, Örebro University, Örebro, Sweden; 9 Turku Bioscience Centre, Department of Life Technologies, University of Turku, Turku, Finland; 10 Faculty of Pharmaceutical Sciences, University of Iceland, Sturlugata 8, 102 , Reykjavik, Iceland; 11 Department of Dermatology, CDL SKINMAGINE, Medical University of Vienna, Währinger Gürtel 18-20, Vienna, 1090 Austria; 12 Department of Translational Genomics, NUTRIM, Maastricht University, Maastricht, The Netherlands; 13 Aston University & Aston Institute for Membrane Excellence, Aston Triangle, Birmingham, B4 7ET UK; 14 Faculty of Medical Sciences, Goce Delcev University, Krste Misirkov, 10A, 2000, Stip, 2000 North Macedonia; 15 Systems Immunity Research Institute, School of Medicine, Cardiff University, Cardiff, CF14 4XN UK; 16 Maastricht MultiModal Molecular Imaging Institute (M4i), Maastricht University, Maastricht, The Netherlands; 17 Centre of Research Excellence in Nutrition and Metabolism, Institute for Medical Research, National Institute of Republic of Serbia, University of Belgrade, Dr. Subotica 4, 11129 Belgrade, Serbia; 18 Metabolomics and Proteomics Laboratory, Clinical Research Centre, Medical University of Bialystok, Marii Skłodowskiej-Curie 24 A, 15-276 Bialystok, Bialystok, Poland; 19 Mass Analytica SL, Av. Cerdanyola 92-94, 08173 Sant Cugat del Vallés, Spain; 20 REQUIMTE, University of Porto, Porto, Portugal; 21 Medical School, Aston University, Birmingham, UK; 22 Department of Marine Microbiology and Biogeochemistry, NIOZ Royal Netherlands Institute for Sea Research, Texel, The Netherlands; 23 Department of Chemistry, CESAM – Centre for Environmental and Marine Studies, University of Aveiro, Campus Universitário de Santiago, 3810-193 Aveiro, Portugal; 24 Center of Membrane Biochemistry and Lipid Research, University Hospital and Faculty of Medicine Carl Gustav Carus of TU Dresden, Tatzberg 47/49, 01307 Dresden, Germany

**Keywords:** Lipidomics, Epilipidome, Collaborative network, COST action

## Abstract

**Background:**

Lipidomics and its branch epilipidomics are rapidly advancing fields that explore the roles of native and modified lipids (e.g., oxidized, nitrated and halogenated lipid species), respectively, in biological systems. Dysregulation of lipid metabolism and signaling contributes to numerous diseases, including cardiovascular, metabolic, neurodegenerative, and inflammatory conditions. However, multiple challenges, including lack of standardization, limited data integration, and poor clinical translation, hinder progress. To address these, the COST Action EpiLipidNET (CA19105) established a pan-European network fostering collaboration across disciplines to accelerate lipid science and its application to health and disease.

**Aim of review:**

This review outlines the achievements of EpiLipidNET over its four-year duration, highlights key scientific contributions across five thematic working groups, and presents the future direction of its ongoing activities. The aim is to demonstrate how a collaborative, interdisciplinary framework can catalyze innovation in lipidomics and epilipidomics, enhance methodological harmonization, support early-career researchers, and bridge the gap between basic science, clinical translation, industry, stakeholders, and public engagement.

**Key scientific concepts of review:**

EpiLipidNET structured its networking activities around (i) harmonization of analytical workflows, (ii) development of epilipidomics tools and data integration strategies, (iii) translational studies for clinical lipid biomarkers, (iv) investigation of lipid signaling mechanisms, and (v) dissemination and outreach. The network supported over 460 members globally, launched multiple training schools and scientific missions, produced 110+ publications, and fostered new initiatives in endothelial membrane lipidomics, food lipidomics, plant and algae lipids, and redox lipid biology. Its integrative approach sets a foundation for continued progress toward precision medicine and sustainable health interventions through lipid science.

## Introduction

**Fig. 1:**
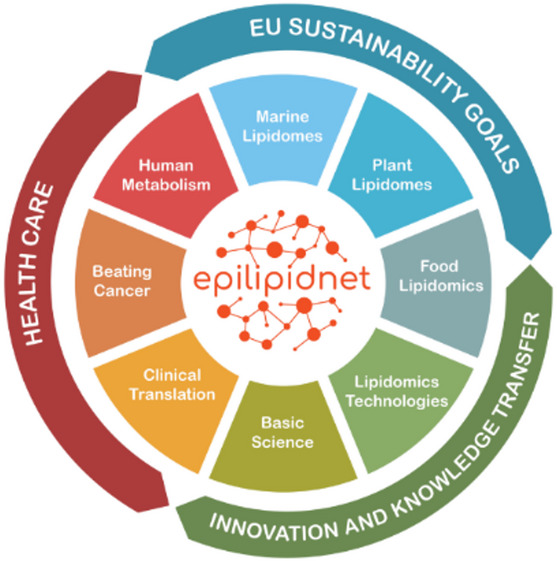
Overview of the primary objectives of the EpiLipidNET community

The EpiLipidNET was a multidisciplinary pan-European scientific network established in 2020 and funded by the European Cooperation in Science and Technology (COST) program (CA19105). EpiLipidNET aimed to advance the fields of lipidomics and epilipidomics by fostering collaboration and active networking among researchers, clinicians, and industry partners. Through its integrative and interdisciplinary framework, EpiLipidNET sought to bridge fundamental and applied lipidomics research, and accelerate discoveries that would contribute to precision medicine, therapeutic innovations, and improve healthcare outcomes. Its primary objectives included expanding lipid research, developing and disseminating novel methodologies for lipid analysis, and translating basic research towards clinical applications for disease prevention, diagnosis, and treatment as outlined in Fig. 1.

Lipids are fundamental biomolecules involved in structural, metabolic, and signaling processes. They serve as essential components of cellular membranes, mediate intracellular communication, and regulate immune responses (Fernandis & Wenk, 2007; O’Donnell et al., 2019). Lipid metabolism dysregulation is implicated in numerous pathological conditions, including cardiovascular diseases, obesity, diabetes, neurodegenerative disorders, and cancer (Leiherer et al., 2022; Hilvo et al., 2020; Tan et al., 2022; Wang et al., 2022; He et al., 2025; Tsap & Shcherbata, 2025; Wolrab et al., 2019, 2022; Zhao et al., 2025). Non-enzymatic and enzymatic modifications of lipids, also referred to as the epilipidome, further contribute to the regulation of cellular and physiological functions as well as disease progression by altering cellular homeostasis and immune function (Penkov et al., 2024). Lipidomics, a rapidly advancing omics field, focuses on characterizing the lipidome, elucidating lipid metabolic pathways, and identifying disease-specific lipid biomarkers. Epilipidomics extends this research by investigating structurally modified lipids, such as oxidized and nitrated species. Technological advancements in mass spectrometry (MS) have boosted lipidomics research by enabling the annotation and quantification of diverse lipid species. However, as with any fast-developing omics field, current challenges in (epi)lipidomics include poor harmonization of analytical workflows, data processing, and integration strategies, as well as lacking proof of readiness for clinical translation. To tackle these challenges in an interactive and collaborative way, the EpiLipidNET network was structured into five Working Groups (WGs), each with a specific focus and aims:

- **WG 1** on **Harmonization of Lipidomics Workflows** aimed to review and establish standardized analytical protocols for comprehensive lipidome coverage across different species, including humans, mammals, invertebrates, algae, plants, and bacteria.
- **WG 2** on **Epilipidomics Analysis and Data Integration** focused on the development of analytical strategies for modified lipids, optimizing MS techniques, and bioinformatics solutions.
- **WG 3** on **Clinical Applications of (Epi)Lipidomics** addressed the translational potential of lipid biomarkers, evaluated their analytical and clinical performance, aiming to facilitate the implementation of lipidomics in clinical and laboratory medicine practice.
- **WG 4** on **Lipid Signaling and Mechanisms of Action** investigated signaling pathways mediated by native and modified lipids, lipid-protein interactions, and their functional implications in cellular and organismal metabolism.
- **WG 5** on **Dissemination and Outreach** was devoted to knowledge transfer, educational initiatives, and public engagement through scientific publications, training programs, and collaborative events.

From the beginning, EpiLipidNET closely collaborated with the leading consortia in the field of lipidomics, including LIPID MAPS, the International Lipidomics Society, the Lipid Standards Initiative, and the Metabolomics Society, among others, fostering a synergistic approach to lipid research. Over four years of active COST funding support (2020–2024), EpiLipidNET united over 460 researchers by facilitating knowledge exchange through conferences, virtual meetings, collaborative research projects, and mobility programs such as Short-Term Scientific Missions (STSMs) and Inclusiveness Target Country (ITC) conference grants. Here, we highlight some of the EpiLipidNET achievements and outcomes over the period 2020–2024 and provide an outlook on future activities of the network and ways to keep engaging with the community.

## The epilipidnet in numbers: what have we achieved?

EpiLipidNET was launched in 2020 as a pan-European, multidisciplinary network with 76 initial members. Launched during the COVID-19 pandemic, the expansion of the community and the development of a functional network emerged as major challenges, as most COST networking instruments were designed to support in-person collaborations and events. EpiLipidNET quickly adapted by hosting virtual events, facilitating the establishment of new partnerships and collaborations, and strengthening of existing ones. This flexibility proved beneficial, allowing both virtual and in-person activities to thrive throughout the entire duration of the initiative. Thanks to its open and inclusive nature, EpiLipidNET has expanded into a global network within just four years, now comprising over 460 members from 52 countries across six continents. The network maintains a strong gender-balance, with 59% female and 41% male members, and 43% of its participants represented by Young Researchers and Investigators (YRIs).

Following the main principle of COST, EpiLipidNET invested in numerous networking activities including annual action-wide conferences, satellite sessions at major symposia and international scientific meetings in the lipid research field, thematic workshops, webinars, and roundtable discussions. To promote knowledge dissemination and exchange, EpiLipidNET provided multiple conference travel grants, and supported inter-laboratory research stays and training schools.

### Action-wide conferences

Overall, EpiLipidNET hosted six action-wide conferences, including 3 online and 3 in-person events, in Portugal, France, and Germany, each attracting on average 150–200 attendees. Each action-wide event included several key components in the program: the YRI committee organized half-day sessions featuring presentations by early-career researchers as well as talks on broader professional development topics. Notable examples include “Imposter phenomenon: definitions and remedies in a research environment” delivered by Dr. Vvedenskaya from the DragonFly non-profit organization (https://dragonflymentalhealth.org/discrete-programs/) dedicated to mental health in academia, “How not to lie with charts” by data visualization expert Dr. Jambor, and a panel discussion “Where do we go next? Career paths, chances and obstacles in the lipidomics field”, including esteemed members from the network. Furthermore, in addition to the regular scientific sessions with oral and poster presentations, each in-person event hosted a roundtable discussion with representatives of industrial partners involved in the network. EpiLipidNET members from technology transfer units, companies specialized in synthesis of lipid standards, software tools, major mass spectrometry vendors, lipidomics services, and representatives of cosmetic industries shared their experiences and perspectives. These discussions provided an interactive platform for the exchange of opinions, views, needs, and ideas, bringing academia and commercial sectors closer to each other. Equally important, the in-person meetings held across three different European countries provided participants, particularly young researchers, with valuable opportunities to experience diverse cultural and academic environments. This exposure enriched their professional development and fostered a greater sense of international collaboration within the network.

### Involvement in specific sessions at major international events

In addition to the action-wide conferences, EpiLipidNET actively supported dedicated sessions and satellite meetings at large international symposia and conferences. Highlights include a workshop on “Clinical Lipidomics” at the 18th Annual Conference of the Metabolomics Society (Valencia, 2022), an EpiLipidNET session at the 11th International Singapore Lipid Symposium (Singapore, 2023), two satellite symposia “Multimodal Imaging of Stress and Inflammation” and “The Metabolome and the (Epi-)lipidome in adaptation to stress” at Society of Free Radical Research Europe (Vienna, 2023) and many others. Overall, with 60 + online and in-person meetings and workshops, EpiLipidNET not only significantly contributed to the dissemination and knowledge transfer, but also to the community-building in the field of lipid research and lipidomics.

To support the dissemination of scientific results obtained by its members, EpiLipidNET awarded a total of 62 conference grants to young researchers from ITC countries. These grants enabled their participation in 38 international scientific conferences across the globe and offered the recipients a unique opportunity for presenting their work, through oral communications or posters, and to engage with the leading experts from the scientific community. Importantly, many of the EpiLipidNET participants were distinguished with awards for best posters and oral communications, highlighting the outstanding quality of the research.

### Exchange between laboratories to innovate together

Another important and highly successful networking tool provided by the COST were STSM grants. EpiLipidNET supported 60 PhD students, postdoctoral researchers, junior investigators and laboratory technicians, enabling them to visit institutions or laboratories in other countries. The STSM grants supported initiatives to foster collaboration, promote knowledge exchange, enhance researchers’ skills, and contribute to scientific progress, supporting research aligned with action goals. Several of these STSMs carried out under the EpiLipidNET umbrella resulted in peer-reviewed publications, demonstrating the scientific value and collaborative strength of these short-term exchanges. For example, the STSM between the University of Perugia and the University of Aveiro enabled the development of a workflow for analyzing phosphatidylinositol modifications and their nitroxidative susceptibility (Bonciarelli et al., 2023), and application of advanced computation tools to study fatty acid oxidation disorders using dried blood spots (Guerra et al., 2025) and contributed to strengthening the established collaboration. An STSM between Friedrich-Alexander-Universität Erlangen-Nürnberg and the University of Montpellier led to the development of derivatization strategies for aldehydic fatty acids (Zartmann et al., 2025). An STSM linking the University of Belgrade and Aristotle University of Thessaloniki enabled the lipidomic analysis of metabolic syndrome in a rat model (Petrovic et al., 2024). An exchange between CEMBIO and Helmholtz Munich led to an advanced MS/MS-based characterization of glycerophospholipids in a reference plasma material (Martínez et al., 2025). A particularly fruitful collaboration between Trinity College Dublin and the University of Aveiro demonstrates the potential of STSMs to initiate broader research outputs, here, a single exchange led to the publication of two independent studies exploring the lipidomic effects of contact allergens in keratinocytes, focusing on fragrance hydroperoxides and terpene-induced lipid peroxidation (Moore et al., 2025a, b). Finally, the University of Graz and TU Dresden investigated the role of phospholipase A2 in processing oxidized phospholipids (Jokesch et al., 2025a, b). In addition to scientific publications, STSMs have played a significant role in career development, specifically for YRIs with respect to future employment, and involvement in scientific societies and editorial boards.

### Support for young researchers

A key focus of EpiLipidNET was the empowerment of early-career researchers via educational and training activities. To this end, the network has (co)organized three training schools aimed at building the expertise of early-career researchers. These included the “LIPID MAPS Spring School 2021” (online), “Lipid Metabolism: from Biochemistry to Clinical translation” (Dresden, 2022), and “Lipid Pathways and Network Analysis” (Maastricht, 2024). Each school included lectures by world-renowned scientists and hands-on sessions on advanced lipidomic technologies and bioinformatics, equipping participants with cutting-edge skills.

EpiLipidNET placed YRIs at the heart of its activities, offering leadership opportunities and actively organizing YRI-driven initiatives. These included dedicated YRI workshops and social gatherings during the six EpiLipidNET conferences, an online skilling workshop “Science meets design” for effective scientific presentations delivered by Dr. Tortorella, a contest for innovative ideas in lipidomics awarded with STSMs grants, a series of interviews answering the question “Why Did You Join EpiLipidNET?”, and the EpiLipidNET Discord server, thus boosting opportunities to actively contribute to the network. Specific activities for YRI training and capacity building organized within EpiLipidNET allowed a dedicated community to be built, enhanced professional expertise, and actively supported career development, putting young scientists in the center role in shaping the network’s future.

### Communicate actively within and for the epilipidnet community

To disseminate EpiLipidNET research, the network actively used social media platforms (LinkedIn, X), established a dedicated website (https://www.epilipid.net), and distributed newsletters to all subscribers with the aim of updating members about ongoing activities. An important feature of the EpiLipidNET website is the online ExpertMap, available to any registered member. This tool lists areas of expertise in both analytical solutions and biological questions. The ExpertMap serves as an online networking tool, where EpiLipidNET members can find collaboration partners and experts in specific areas of interest. The research outcomes of EpilipidNet were substantial, with 110 articles published in peer-reviewed journals. A Virtual Special Issue containing 16 articles was organized in collaboration with the publisher Elsevier (Zartmann et al., 2025; Jokesch et al., 2025a, b; Spickett et al., 2025a, b; Kose et al., 2024; Biagini et al., 2025; Halasz et al., 2024; Chang et al., 2025; Jouhet et al., 2024; Parchem et al., 2024; Puig et al., 2025; Ten-Doménech et al., 2024; Mateo-Marín & Alves-Bezerra, 2024; Martínez et al., 2024; Hajeyah et al., 2024; Company-Marín et al., 2025; Varandas et al., 2024).

### Introducing everyone to the world of lipids

**Fig. 2:**
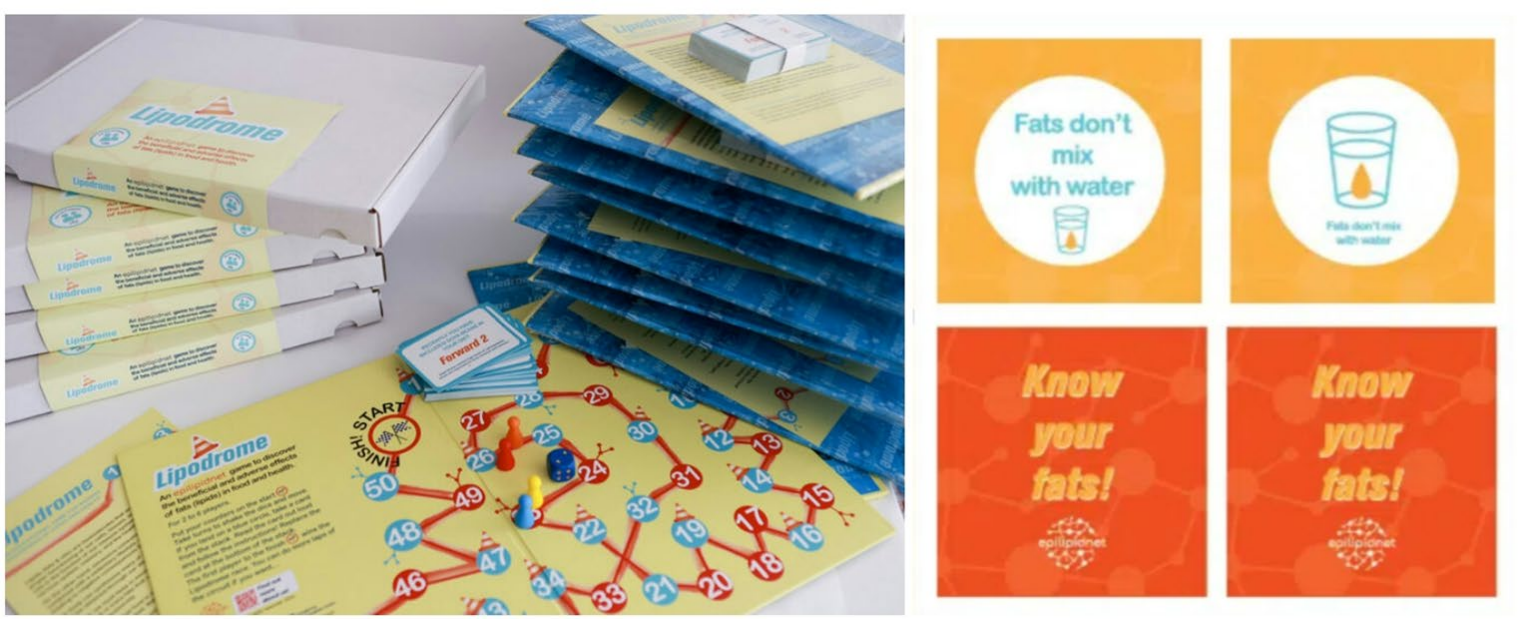
“Lipodrome” (left) and “Know your fats” (right) are part of the gaming suite developed within the framework of EpiLipidNET to foster lipid-related dissemination activities

Furthermore, fostering communication not only among EpiLipidNET researchers but also with society and key stakeholders has been a central priority for the network. To raise awareness about the role of lipids in health and disease, a video (co-organized by YRIs) and three educational games were developed and distributed across multiple countries as part of a pan-European outreach strategy. The gaming suite included: (i) “Lipodrome” (board game, Fig. 2, left) (Spickett et al., 2025a, b) and (ii) “Know Your Fats!” (memory game, Fig. 2, right) (Tortorella et al., 2025) for general audiences, and (iii) “Burn Your Fat” (Milonaitytė & Kuda, 2024; IPHYS, 2025) for high school and undergraduate students. These games have been presented at EpiLipidNET events and are currently being translated into multiple languages. They have become a popular educational toolset at a wide range of events, including citizen-oriented activities such as the “Long Night of Science,” targeted educational workshops in schools, and as icebreakers at kick-off meetings of research networks. Importantly, the games are published under the CC BY-NC-SA 4.0 license, enabling free use, adaptation, and further development for non-commercial purposes.

A major milestone of the dissemination efforts was the exploitation event titled “Bridging Lipids to Society” held in Brussels (September 2024), where EpiLipidNET findings were shared with policymakers, members of the European Parliament, and other stakeholders. Discussions focused on lipids in mental health, health challenges, and educational outreach. This event was featured in a COST press release, “Bridging Lipids and Society”. All the outreach activities significantly contributed to increasing public awareness and understanding of the importance of lipids. Table 1 provides a summary of the EpiLipidNET’s achievements.

**Table 1:** EpiLipidNET in numbers. A summary of the outcomes of the collaborative network

Membership 460 + members from 52 countries and 6 continents Gender balance: 59% female | 41% male 43% of members are YRIs
Conference and Workshop organization and co-organization 6 conferences: 3 online + 3 on-site Sessions and satellite meetings at international conferences • 9th International Singapore Lipid Symposium, online, 2021 • 18th Annual Meeting of the Metabolomics Society, Valencia, Spain, 2022 • Lipid Forum, Vienna, Austria, 2023 • “Nuclear Lipids in Health and Disease” at the Wilhelm Berhard Workshop on the Cell Nucleus, Prague, Czech Republic, 2023 • 11th International Singapore Lipid Symposium, Singapore, 2023 • Annual Meeting of Society for Free Radical Research – Europe (SFRR-E), Vienna, Austria, 2023 • Marine Plants and Algae Lipidomics at the 32nd Nordic Lipid Forum Symposium, Turku, Finland, 2024 On-site workshops: • Mass spectrometry data processing and molecular networking using MZMINE, GNPS, and SIRIUS, Prague, Czech Republic, 2021 • Chemometric Analysis in Lipidomics, Perugia, Italy, 2022 • Hackathon – Curation of Model Organism Lipidomes, Neuherberg, Germany 2023 • “Lipids and Cell Plasticity: Implications for Tissue Regeneration, Cancer, and Immunology, Aveiro, Portugal, 2024 • Workshop “Human milk lipids analysis: sampling, storage, isolation and determination methodology”, Gdansk, Poland 2024 + 60 online workshops and WG meetings
Training schools 1 online Training School • LIPID MAPS Spring School 2021 2 on-site Training Schools: • Lipid Metabolism: From Biochemistry to Clinical Translation, Dresden 2022 • Lipid Pathways and Network Analysis, Maastricht 2024
Grants 62 ITC Grants: participation in 38 different international conferences, 3 of which in Singapore, 1 in Japan, 1 in USA, 1 in Australia and 1 in Canada 60 STSM Grants: held in 18 different countries, 3 of which in Japan and the USA; 29 grantees from ITC | 31 grantees from non-ITC
Publications 118 publications https://www.epilipid.net/dissemination-outreach/
Special issue EpiLipidNET Virtual Special Issue on Analysis and Biological Importance of Lipids and Modified Lipids, Elsevier https://www.sciencedirect.com/special-issue/10CJCNZ9MHC 16 publications: 2 Reviews and 14 original articles in 7 journals
Projects 19 submitted project proposals (17 to European programs and 2 to national calls)
Website https://www.epilipid.net/
Videos Welcome to EpiLipidNET! https://www.epilipid.net/dissemination-outreach/ EpiLipidNET: Memories from 2020–2024 https://www.epilipid.net/wp-content/uploads/2025/03/EpiLipidNET-Video_Memories-from-2020-to-2024.mp4 Boardgame session at EpiLipidNET general action meeting in Toulouse, 2023 https://www.epilipid.net/wp-content/uploads/2025/09/Playing-games.mp4 Interview video series “Why Did You Join EpiLipidNET?” https://www.epilipid.net/wp-content/uploads/2025/09/Interviews.mp4
Educational Games https://www.cost.eu/scicomm-epilipidnet/ https://www.lipidmaps.org/resources/education/resources Lipodrome: https://data.mendeley.com/datasets/f6hk39ztkd/1 (Mendeley Dataset metrics Views 99 Downloads 35 on 29.9.2025) Know Your Fats! https://data.mendeley.com/datasets/42twmxm275/1 (Mendeley Dataset metrics Views 72 Downloads 15 on 29.9.2025) Burn your fat https://github.com/IPHYS-Bioinformatics/Burn_your_fat
Exploitation Event EpiLipidNET Exploitation Meeting “Bridging lipids and society”, Brussels, September 2024 https://www.cost.eu/bridging-lipids-epilipidnet/

## EpiLipidNET: challenges and success stories in lipid science and collaboration

The EpiLipidNET COST action has been a driving force in uniting lipid researchers across Europe and beyond, fostering breakthroughs in lipidomics and epilipidomics. It is important to note that the establishment and maintenance of such a large research network inevitably involve certain challenges. Although the COST grant supports a part-time administrative position, essential for ensuring effective and timely financial management, the majority of organizational efforts, ranging from strategic guidance to day-to-day operations, rely entirely on the enthusiasm and substantial time commitment of network members. To support the effective operation of EpiLipidNET, a Core Group was established as the main decision-making body. The Core Group comprised the Chair, Vice-Chair, Working Group leaders and co-leaders, and the heads of several committees, including the Grant Awarding Committee (covering STSM and ITC grant proposals), the YRI Committee, the Industrial Coordination Committee, and the Science Communication Manager. This group met monthly throughout the four-year duration of EpiLipidNET, with each member dedicating a substantial amount of time to the success of the Action. Together with other members, including participants in the STSM and ITC grant review panels and local organizers of EpiLipidNET Annual Meetings, Training Schools, and conference sessions, their commitment, engagement, willingness to share responsibilities, and genuine enthusiasm made this CA a success.

Based on these experiences, several considerations may be useful for researchers planning similar COST Actions. During the preparation phase, it is important to maintain a clear and consistent focus on the scientific and societal need for the proposed Action throughout the planning and writing process, explicitly articulating how advances in the field (in this case lipidomics) can translate into tangible medical, nutritional, and industrial benefits. This broader framing is essential not only for demonstrating impact beyond academia but also for engaging non-academic stakeholders from an early stage. Careful attention should also be given to achieving a balanced consortium that combines disciplinary breadth with complementary academic and non-academic expertise. During implementation, establishing clear governance structures and communication channels early on can help manage rapid network growth and maintain effective coordination. Sustained engagement of participants requires regular opportunities for meaningful contribution, while long-term sustainability benefits from early consideration of post-Action collaborations and funding pathways. Finally, engaging industrial partners often requires tailored communication and clear demonstration of mutual benefit, underscoring the importance of continued outreach beyond the academic community.

By creating an inclusive, collaborative environment, EpiLipidNET has empowered scientists to advance knowledge, standardize methodologies, and engage with society. The summary below showcases only some of many accomplishments of EpiLipidNET members.

The networking activities within WG1 addressed several critical points in harmonization of lipidomics workflows, ranging from educational online workshops “How to do…” presented by analytical experts in different areas to organizing the first-in-class hackathon on “Model organism lipidomes”. WG1 network specifically addressed the issue of lipid annotations using diverse types of identifiers across the field, a key challenge which limits data integration efforts by the lipidomics community (Witting et al., 2024). In the fast-developing field of (epi)lipidomics, multiple bioinformatics tools are being developed to facilitate different aspects of data processing. The diversity of available tools can be overwhelming to the end users, so to guide evidence-based selection of the software in the field on MS-based lipid analysis, WG1 and WG2 in close collaboration with LIPID MAPS developed a Lipidomics Tools Guide (Ni et al., 2023). 

WG2 expanded on this initiative by producing a comprehensive review on the bioinformatics tools available for the analysis of epilipidomics datasets (Damiani et al., 2023). Compared to conventional lipidomics, both the analysis and data processing of modified lipids remain in their early stages. WG2 members made a significant contribution to advancing this emerging field (Criscuolo et al., 2022; Neves et al., 2022; Ferreira et al., 2022; Elloumi et al., 2024).

WG3 on clinical translation of (epi)lipidomics addressed the important question of intra- and inter-individual variability in the human lipidome (Nguyen et al., 2025). Furthermore, a multi-center study to investigate the impact of microsampling of whole blood and plasma on the coverage of detected lipid species was initiated. This study utilized a comprehensive range of commercially available kits and aimed to assess the reliability, reproducibility, and lipidomic coverage of different microsampling approaches across 19 participating laboratories from Europe, Canada, and Singapore.

WG4 on lipid signaling and mechanisms of action contributed to public knowledge dissemination of specific metabolic- and signaling pathways through the collaboration with WikiPathways, the Lipids Portal on WikiPathways (https://lipids.wikipathways.org/) (Agrawal et al., 2024), a well-established open-access, community-driven resource that enables broad dissemination of curated lipid-related signaling and metabolic pathways, facilitated by continuous expert curation, and supporting reproducible and transparent pathway knowledge for the wider scientific community. The EpiLipidNET website serves as a central hub for the community, highlighting the extensive outcomes of WG4 collaborations. Many of these collaborative efforts have proved so impactful that they continue independently beyond the official funding period, demonstrating the lasting influence and sustainability of the network’s initiatives.

## The future of the epilipidnet

Given that COST Action support is limited to four years, a further major challenge arising from the success of EpiLipidNET has been maintaining momentum and ensuring the sustainability of the established network. Although united by a shared interest in lipids and their modified forms, EpiLipidNET members represent a highly multidisciplinary community. To address the broad range of interests within the network, a series of online events entitled “All4Lipids” was organized during the final year of the Action and after, to foster stronger collaboration with well-established scientific societies, ensuring the long-term sustainability and wider impact of EpiLipidNET. These events invited representatives of various scientific societies, such as the Metabolomics Society, the International Lipidomics Society, Euro Fed Lipids, GERLI, and the Society for Free Radical Research, among others, to present their activities to EpiLipidNET members.

Furthermore, although the formal COST Action has officially concluded, the EpiLipidNET community remains highly active through newly undertaken actions aimed at maintaining and further developing its activities and future initiatives enduring scientific impact. These include regular thematic virtual webinars, support for newly approved and emerging COST Actions (e.g., CA24138 EU-RESOLVE and others), the development of new collaborative project proposals, participation in ring trials, and the preparation of joint position papers. In parallel, EpiLipidNET continues to strengthen its ongoing collaborations within established Interest Groups, which continue to involve the wider community, attract new members, and expand into newly emerging thematic areas.

### Specific interest groups (IGs) for focus discussions

The **EndotheliOme IG** is a cross-disciplinary collaborative network of researchers with background on cell biology, analytical chemistry, membrane biophysics, biostatistics, cell imaging, and computational simulations, gathered to advance the field of endothelial membrane biology. In light of the emerging role of endothelial plasma membrane in vascular mechano-sensing triggering downstream signaling pathways (Yamamoto & Ando, 2018), the IG already performed the in-depth analysis of plasma membrane vesicles isolated from primary human umbilical vein endothelial cells grown in glucose-stressed conditions. On-going computational simulations aim to bring insights into the impact of lipid remodeling on endothelial membrane biophysics and how this reflects on lipid-protein interactions involved in sugar and lipid metabolism. With interests on the role of (oxy)lipids to membrane organization, trafficking and signalling (Kose et al., 2024; Reis & Dias, 2024), the IG aims to share findings, tools and techniques with a wider audience through the EndotheliOme webinar series and foster discussions on the importance of lipids to vascular biology.

The **Food Lipidomics** IG brings together over 40 researchers from diverse academic and industry backgrounds, united by a shared commitment to advancing the field of food lipidomics. Food lipids have received growing attention throughout the EpiLipidNET action, not only for their nutritional and functional significance, but also for their impact on human health and their critical role in ensuring food quality, traceability, and safety. Through interdisciplinary collaboration, the Food Lipidomics IG has played a central role in identifying research priorities and addressing methodological challenges, culminating in a high-impact review paper that synthesizes key findings and sets the challenges for future research (Tietel et al., 2023). The IG has also contributed to the dissemination of impactful findings, notably through the thematic press release “Lipids are Good for Human Health” (https://www.cost.eu/introducing-lipids-epilipidnet/), which helped raise awareness of the nutritional and societal importance of lipid research. Committed to long-term impact, this IG plans to continue fostering collaboration, support early-career researchers, and drive innovation in lipid analysis and its application towards sustainable food systems.

Since the launch of EpiLipidNET, the **Algae and Plant Lipidomics** IG has been actively advancing research and promoting knowledge exchange in the field of algal and plant lipidomics. With over 30 core members, the IG collaboratively authored a comprehensive review that outlines key analytical methodologies, explores the lipid composition of algae and plants, and highlights the broader societal relevance of this research (Jouhet et al., 2024). After the conclusion of EpiLipidNET, the group initiated a bi-monthly webinar series showcasing the work of diverse research teams in the field, with a special emphasis on presentations by early-career scientists.

The activities of WG2, which focused on analysis of epilipids, including oxidized and nitrated species, will be continued via the **Redox and Epilipidomics IG**. This IG will work in a close collaboration with thematic societies and groups in the field of redox biology, including the Internation 4-Hydroxynonenal Club and Society of Free Radical Research Europe. Over the years, exciting research in the field of lipid modifications and especially lipid (per)oxidation brought epilipidomics to the forefront of biomedical research. Development of new technologies allowed identification of the role of oxidized/oxygenated lipids in the pathophysiology of numerous metabolic and degenerative disorders, including cancer (Berndt et al., 2024). Recent discoveries placed lipid peroxidation at the crossroad of cell fate decisions, with lipid oxidation acting as a key executor of ferroptotic cell death. The role of oxygenated lipids in both acute and chronic inflammatory disorders is substantiated by key discoveries in the field of oxylipin signaling and metabolism (Schebb et al., 2025). Moreover, protein modifications by electrophilic products of lipid peroxidation act as signaling cues in regulation of complex biological functions. The major aim of the Redox and Epilipidomics IG is to provide a discussion and networking platform for researchers interested in developing novel methodologies for identification, annotation, and quantification of modified lipids, as well as addressing the biological effects of modified lipids in physiological and pathological settings.

### Building networks beyond epilipidnet

EpiLipidNET members will continue their close collaboration with members of other lipid-oriented communities. The strong links with LIPID MAPS have been demonstrated over four years of EpiLipidNET existence via multiple activities ranging from organization of training events and the development of the Lipidomics Tools Guide to revision of lipidomes of model organisms. Current work on the generation of spectral databases (i.e., PartialDB) for partially assigned lipids initiated at one of the EpiLipidNET meetings is providing a tool for the analysis of “unknown” lipid species, boosting the coverage of natural lipidomes (https://www.lipidmaps.org/databases/partial_db/overview). EpiLipidNET members contributed to the major harmonization efforts undertaken by the International Lipidomics Society (Schebb et al., 2025) and will continue to work together in the frame of various IGs within ILS. The Lipidomics Task group (LipidMet) was initiated within the Metabolomics Society by action members. The aim of the LipidMet Task Group is to bring together the expertise and experiences from both metabolomics and lipidomics fields and unite them under the common umbrella of metabolism research with shared best practices, harmonization and quality control protocols.

Overall, the EpiLipidNET journey over the last four years enabled consolidation of knowledge and expertise in lipid research to the level we hardly could have predicted back in 2020. The power of networking, and an inclusive and welcoming environment created by the EpiLipidNET community, not only boosted the scientific and technological development of lipid research but also resulted in a network of close friends and collaborators. However, above all, EpiLipidNET’s dedication to training and educational activities allowed us to develop a cohort of excellent young researchers who are already becoming leaders in the field of (epi)lipidomics.

## Data Availability

No datasets were generated or analysed during the current study.
